# Accuracy and variability of locoregional staging in T1 rectal cancer: nationwide multicentre cohort study

**DOI:** 10.1093/bjsopen/zrag003

**Published:** 2026-03-31

**Authors:** Maria Daca-Alvarez, Cristina Manzotti, Diana Zaffalon, Isabel Portillo, Luis Bujanda, Inés Gil-Lasa, Gemma Ibañez-Sanz, Xavier Sanjuan, Alberto Herreros-de-Tejada, Inmaculada Salces, Lara Aguilera, Marta Ponce, Ángeles Pizarro, David Barquero, Ignasi Puig, Pilar Diez Redondo, Fernando Martínez de Juan, Victor Jair Morales, Marco Alburquerque, Salvador Machlab, Angel Ferrandez, Beatriz Peñas, Alvaro Díaz-González, Lluïsa Sargatal, Rodrigo Jover, Luis Hernandez, Alberto Pérez Pedrosa, Eva Musulen, Goretti Hernandez, Marita Trelles, Akiko Ono, Jorge Lopez Vicente, Raquel Bravo, Juan R Ayuso, Angels Ginés, Karmele Saez de Gordoa, Miriam Cuatrecasas, Maria Pellisé, EpiT1 Consortium, G Casanova, O Murcia, M Jimeno, N Ascon, F Balaguer

**Affiliations:** Gastroenterology Department, Hospital Clinic of Barcelona, Clinic Institute of Digestive and Metabolic Disease (ICMDIM) of Barcelona, Barcelona, Spain; Centro de Investigación Biomédica en Red en Enfermedades Hepáticas y Digestivas (CIBEREHD), Institut d'Investigacions Biomédiques August Pi i Sunyer, Barcelona, Spain; Gastroenterology and Digestive Endoscopy Unit, Azienda USL-IRCCS Reggio Nell'Emilia, Reggio Emilia, Italy; Gastroenterology Department, Consorci Sanitari de Terrassa, Barcelona, Spain; Dirección General de Osakidetza-Servicio Vasco de Salud, Instituto de Investigación BioBizkaia, Barakaldo, Spain; Department of Gastroenterology, BioGipuzkoa Health Research Institute, Centro de Investigación Biomédica en Red de Enfermedades Hepáticas y Digestivas (CIBERehd), Universidad del País Vasco (UPV/EHU), San Sebastián, Spain; Department of Gastroenterology, BioGipuzkoa Health Research Institute, Centro de Investigación Biomédica en Red de Enfermedades Hepáticas y Digestivas (CIBERehd), Universidad del País Vasco (UPV/EHU), San Sebastián, Spain; Medical Oncology, ICO Institut Català d'Oncologia, L'Hospitalet de Llobregat, Barcelona, Spain; Hospital Universitari de Bellvitge, ONCOBELL Program, Bellvitge Biomedical Research Institute (IDIBELL), Barcelona, Spain; Hospital Universitari de Bellvitge, ONCOBELL Program, Bellvitge Biomedical Research Institute (IDIBELL), Barcelona, Spain; Department of Gastroenterology, Puerta De Hierro University Hospital, Madrid, Spain; Gastroenterology Department, Hospital Universitario 12 de Octubre, Madrid, Spain; Gastroenterology Department, Hospital Vall D'Hebron, Barcelona, Spain; Gastroenterology Department, Hospital Clinico Valencia, Valencia, Spain; Gastroenterology Department, Hospital Universitario Virgen del Rocío, Sevilla, Spain; Gastroenterology Department, Hospital Sant Joan Despí Moisès Broggi, San Joan Despi, Barcelona, Spain; Digestive Diseases Department, Althaia Xarxa Assistencial Universitària de Manresa, Manresa, Spain; Facultat de Medicina, Universitat de Vic-Central de Cataluña (UVIC-UCC), Vic, Spain; Gastrointestinal Oncology Endoscopy and Surgery (GOES) Research Group, Institut de Recerca I Innovació en Ciències de la Vida I de la Salut a la Catalunya Central (IRIS-CC), Vic, Spain; Gastroenterology Department, Hospital Universitario Río Hortega, Valladolid, Spain; Gastroenterology Unit, Instituto Valenciano de Oncología (IVO), Valencia, Spain; Gastroenterology Department, Hospital General de Granollers, Barcelona, Spain; Gastroenterology Department, Hospital de Palamós, Girona, Spain; Digestive Endoscopy Unit, Institut d‘Investigació I Innovació Parc Taulí I3PT, Parc Taulí Hospital Universitari, Sabadell, Spain; Departament de Medicina, Universitat Autónoma de Barcelona, Barcelona, Spain; Gastroenterology Department, Hospital Clínico Universitario Lozano Blesa, Zaragoza, Spain; Gastroenterology Department, Hospital Universitario Ramón y Cajal, Madrid, Spain; Gastroenterology and Hepatology Department, Clinical and Translational Research in Digestive Diseases Group, Valdecilla Research Institute (IDIVAL), Marqués de Valdecilla University Hospital, Santander, Spain; Gastroenterology Department, Consorci Sanitari de Terrassa, Barcelona, Spain; Servicio de Medicina Digestiva, Hospital General Universitario Dr. Balmis, Instituto de Investigación Sanitaria ISABIAL, Departamento de Medicina Clínica, Universidad Miguel Hernández, Alicante, Spain; Gastroenterology Department, Hospital Santos Reyes Aranda Duero, Burgos, Spain; Gastroenterology Department, Complexo Hospitalario de Ourense, Ourense, Spain; Hospital Univeritari General de Catalunya-Grupo Quironsalud, Insitut de Recerca contra la Leucèmia Josep Carreras, Barcelona, Spain; Servicio de Aparato Digestivo, Hospital Universitario de Canarias, Santa Cruz de Tenerife, Spain; Gastroenterology Department, Hospital de Inca, Islas Baleares, Spain; Gastroenterology Department, Hospital Virgen de la Arrixaca, Murcia, Spain; Gastroenterology Department, Hospital Universitario de Móstoles, Madrid, Spain; Department of Colorectal Surgery, Hospital Clínic of Barcelona, Barcelona, Spain; Radiology Department - CDI, Hospital Clínic of Barcelona, Barcelona, Spain; Gastroenterology Department, Hospital Clinic of Barcelona, Clinic Institute of Digestive and Metabolic Disease (ICMDIM) of Barcelona, Barcelona, Spain; Centro de Investigación Biomédica en Red en Enfermedades Hepáticas y Digestivas (CIBEREHD), Institut d'Investigacions Biomédiques August Pi i Sunyer, Barcelona, Spain; Facultat de Medicina i Ciències de la Salud, Universitat de Barcelona (UB), Barcelona, Spain; Department of Pathology, Centre of Biomedical Diagnosis (CDB), Hospital Clínic of Barcelona, Barcelona, Spain; Facultat de Medicina i Ciències de la Salud, Universitat de Barcelona (UB), Barcelona, Spain; Department of Pathology, Centre of Biomedical Diagnosis (CDB), Hospital Clínic of Barcelona, Barcelona, Spain; Gastroenterology Department, Hospital Clinic of Barcelona, Clinic Institute of Digestive and Metabolic Disease (ICMDIM) of Barcelona, Barcelona, Spain; Centro de Investigación Biomédica en Red en Enfermedades Hepáticas y Digestivas (CIBEREHD), Institut d'Investigacions Biomédiques August Pi i Sunyer, Barcelona, Spain; Facultat de Medicina i Ciències de la Salud, Universitat de Barcelona (UB), Barcelona, Spain

**Keywords:** early stage, staging, endoscopic ultrasound, magnetic resonance imaging

## Abstract

**Background:**

Accurate locoregional staging is critical in the management of T1 rectal cancer and guides organ-preserving strategies. Despite guideline recommendations, the real-world performance of magnetic resonance imaging (MRI) and endoscopic ultrasound (EUS) remains unclear. This study aims to evaluate the use and accuracy of locoregional staging in T1 rectal cancer.

**Methods:**

Retrospective analysis was performed from a nationwide multicentre T1 colorectal cancer cohort (EpiT1 Consortium) between 2007 and 2018 with a 5-year follow-up. Locoregional staging methods included MRI and EUS. Multivariable logistic regression identified factors associated with locoregional imaging. The accuracy of each technique and their combination for T and N staging was assessed.

**Results:**

Among 3161 patients with T1 colorectal cancer, 681 had rectal cancer, of which 424 (62.3%) underwent locoregional staging: 234 (55.2%) with MRI only, 131 (30.9%) with both MRI and EUS, and 59 (13.9%) with EUS only. Factors associated with imaging (MRI and EUS) included management at a referral centre (odds ratio 2.9, 95% confidence interval 1.5 to 5.7), tumour location in the low/middle rectum (odds ratio 3.2, 1.8 to 5.7), suspicion of invasive carcinoma at colonoscopy (odds ratio 2.4, 1.3 to 4.5), and high-risk histological features (odds ratio 3.7, 1.8 to 7.4). MRI accurately staged T in 28.3%, whereas EUS achieved an accuracy of 59% for T staging. Combining modalities overstaged 67.1% of tumours for T staging. N staging was detected with ≤ 16% sensitivity across all strategies; in the surgical group (MRI and/or EUS), overall accuracy was 78%.

**Conclusion:**

Locoregional staging varied widely and was influenced by non-tumour-related factors. MRI and EUS showed modest accuracy, overstaging T, and low sensitivity for N. These findings highlight the need to improve pretreatment evaluation of T1 rectal cancer.

## Teaser

There is no clear consensus on the optimal methods for locoregional staging of rectal polyps suspected to be T1 cancers. This study emphasizes the high variability in clinical practices and limitations of EUS and MRI in real-life clinical settings.

## Introduction

Rectal cancer represents approximately 40–50% of all colorectal cancers (CRCs) and differs significantly from colonic cancer in its molecular, biological, and clinical features, requiring different management strategies^1,2^. Treatment is guided by TNM staging. T1 CRC may be managed with local treatments such as endoscopic resection or transanal surgical excision. Oncological surgery of the rectum is technically challenging, and associated with considerable morbidity and impaired quality of life. Recently, therapeutic strategies focused on organ preservation have emerged, including advanced endoscopic and minimally invasive surgical techniques, together with structured watch-and-wait protocols^3^. These approaches offer curative potential with fewer complications and improved quality of life.

Accurate local staging of rectal cancer is crucial to selecting the appropriate therapeutic strategy. Two primary techniques are used for locoregional staging: endoscopic/endorectal ultrasound (EUS/ERUS) and magnetic resonance imaging (MRI)^3^. EUS/ERUS can assess tumour invasion depth and describe the shape, size, edge, and echogenicity of pararectal lymph nodes up to the iliac vessels^4^. MRI can provide similar information and a broader image of the mesorectum, the entire pelvis, lymph node stations, and the tumour's relationship with the anterior peritoneal reflection and the anal canal, which is vital for both staging and surgical planning^5–8^. Whereas MRI is considered the standard for advanced tumours, consensus on the diagnostic algorithm for early rectal cancer is lacking. A meta-analysis reported comparable overall accuracy, yet ERUS was more sensitive for T staging than MRI (82% *versus* 69%) and another meta-analysis^7^ confirmed that EUS remains the most accurate modality for T staging in early disease. Recent evidence^9^ shows that two-dimensional T2-weighted and diffusion-weighted MRI have limited accuracy for distinguishing T1 from T2 rectal tumours, whereas MRI remains helpful for separating T2 from T3 disease. According to NCCN 2025 guidelines^10^, high-resolution pelvic MRI is recommended for local staging, with endoscopic or endorectal ultrasound reserved for patients in whom MRI is contraindicated, unavailable, or non-diagnostic, particularly for early cT1–T2 lesions. A recent review^11^ emphasizes the challenge of differentiating T1–T2 tumours on MRI, suggesting EUS/ERUS for improved specificity in early-stage cases. Optical diagnosis in centres with advanced endoscopic technologies may aid in distinguishing T1–T2 tumours^12–18^.

In clinical practice, variability exists in the staging and management of early rectal cancer. Factors such as diagnostic equipment availability, specialist expertise, and multidisciplinary team discussions contribute to this variability. Real-world settings can deviate from optimal conditions, impacting the accuracy of staging compared with literature-based evidence. The surgical approach for rectal T1 lesions at high risk for lymph node metastases (LNMs) or local recurrence remains debated, lacking consensus on defining high-risk factors^19–21^. Whereas T1 rectal tumours are typically treated locally, they present diagnostic challenges and can be incorrectly identified macroscopically as invasive cancers. Approximately 21–67% of T1 CRC cases are initially suspected to be invasive cancers that ultimately are confirmed to be non-invasive neoplastic polyps^14,17^.

Given the increasing prevalence of T1 rectal cancers, especially within screening programmes, and the expanding therapeutic options, there is an urgent need to improve the management of these lesions. This multicentre nationwide cohort study aims to analyse the accuracy and the factors associated with locoregional staging of T1 rectal cancers and to estimate the proportion of potentially overtreated patients, addressing the gaps in current understanding and offering insights into the evolving landscape of early rectal cancer management.

## Methods

### Patient selection

A population-based multicentre cohort study involving 33 health centres in 12 states (EpiT1 consortium) from Spain, including all patients who had a histopathological diagnosis of T1 CRC between 2007 and 2018, regardless of treatment received. Exclusion criteria were histology other than adenocarcinoma, hereditary high-risk CRC syndromes, inflammatory bowel disease, synchronous or metachronous CRC in the previous 5 years, or metastatic neoplastic disease at the time of diagnosis. For each patient, 505 variables were evaluated including baseline patient and lesion characteristics, therapeutic procedural details and adverse events, histology, and staging and surveillance procedures. Five-year follow-up outcomes were recorded in an electronic database in AEG (Spanish Association of Gastroenterology) - REDCap® (Research Electronic Data Capture), Vanderbilt University, Nashville, TN, USA. For this analysis, only patients with T1 tumours located in the rectum and with complete information on locoregional staging were included.

This study was conducted and reported in accordance with the STROBE guidelines^22^ (*Appendix S1*).

### Definitions

‘Local treatment’ was defined as mucosal or transmural treatment with endoscopy or transanal endoscopic microsurgery (TEM)/transanal minimally invasive surgery (TAMIS). ‘Oncological surgery’ was defined as any kind of radical surgical treatment that was associated to lymphadenectomy, independent of the specific surgical approach. ‘Oncological adverse outcome’ was defined as the presence of positive lymph nodes at surgical pathology for patients treated with oncological surgery, or any kind of extraluminal recurrence of the disease.

A staging test was defined as any MRI and/or EUS performed between the date of diagnostic colonoscopy and the date of surgery for those patients finally submitted to oncological surgery (primary or secondary) or 1 month after local treatment for those not undergoing oncological surgery. In the latter case, the information was only used for N staging.

Referral centres were defined as a tertiary-care hospital with specialized units and trained experts, where complex or high-risk cases are routinely referred from other hospitals within the health system (*Table S1*).

For the T stage, only overstaging could be assessed, as the database consisted of patients with a known T1 CRC. Patients reported with ‘Tx’ stage, or a non-declared T stage were excluded from the accuracy analysis as it was not possible to establish retrospectively whether they had to be interpreted as having adenomas or as non-assessable.

For the N staging, it was assumed that the healthcare centres involved in the study followed the first version of the ESGAR guidelines after 2013 (first version published in 2013^23^, last update in 2018^9^). Patients with an Nx staging or a non-declared staging in the database were excluded, as these groups could include both patients with missing data on N staging or with an unclear staging after undergoing the imaging modality. Patients who underwent neoadjuvant therapy (NAT), were also excluded, as this could affect the standard to evaluate the accuracy of staging. The N staging defined by EUS or MRI was considered as ‘N0’ if the stage was N0 and ‘N+’ if equal to or greater than N1. For patients who underwent both EUS and MRI, the staging was considered as ‘N0’ if both were N0 and ‘N+’ if at least one stage between EUS or MRI was N+.

For the standard, the presence of at least one positive lymph node in the surgical specimen was considered N+. Lymphovascular invasion, poor differentiation (grading G2/G3), tumour budding (TB 2,3) and combined free margins < 1 mm or unknown were considered as high-risk histological features. Lesions with one of these features were considered as high-risk lesions.

### Statistical analysis

For this analysis, variables were considered from the baseline characteristics of patients, the cancerous lesions, treatment, histology, and follow-up. Statistical analysis was carried out with IBM SPSS Statistics, version 25 (SPSS Inc-IBM Corp., Chicago-Illinois-USA). Descriptive statistics were used to analyse the results using counts and proportions for categorical variables and mean(standard deviation(s.d.)) or median (interquartile range (i.q.r.)) for continuous variables when appropriate. Univariable analysis was carried out with the χ^2^ test for proportions, Student’s *t* test for mean of variables with normal distribution, and Mann–Whitney *U* test (Wilcoxon rank-sum test) for median of variables with non-normal distribution. Multivariate analysis was performed with binary logistic regression on variables with a statistical significance in the univariate analysis and/or clinically relevant.

Two-by-two tables were used to calculate sensitivity, specificity, positive predictive values, and negative predictive values for T and for N stages. As the database consisted of patients with a known T1 CRC, only overstaging could be assessed for the T stage. Mc Nemar’s test was used to compare paired data on sensitivity and specificity of EUS and MRI for N stage considering only data of patients who underwent both imaging modalities. Histopathology was considered the standard when available. In those cases that were treated only locally and not submitted to lymphadenectomy, any recurrence different from endoluminal was assumed as a surrogate of lymph node positivity (see definition of ‘oncological adverse outcome’ above).

## Results

### Baseline characteristic of the cohort

**Fig. 1:**
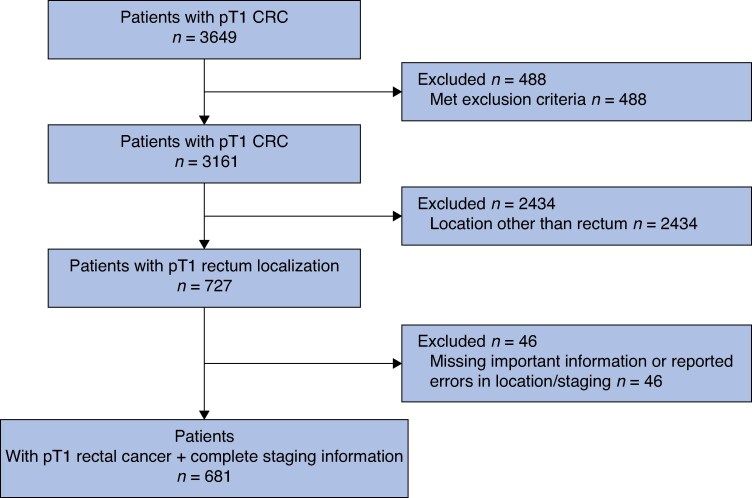
Flowchart selection of patients CRC, colorectal cancer.

**Fig. 2:**
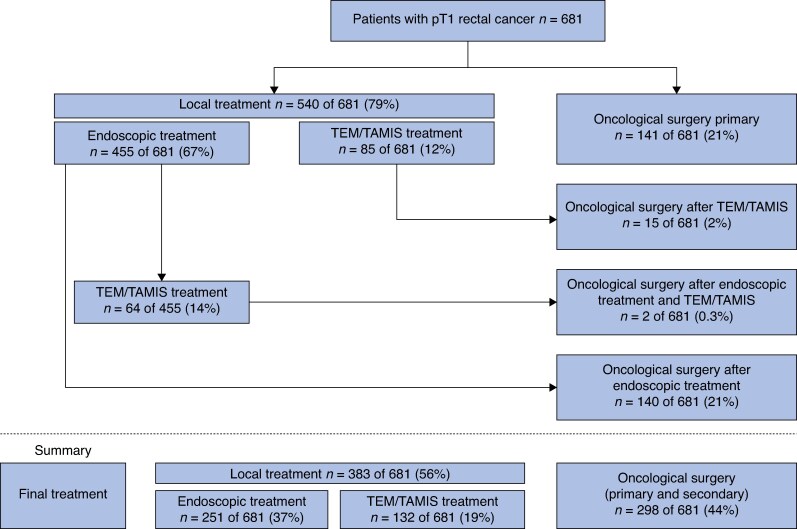
Treatment received for T1 rectal cancer TEM, transanal endoscopic microsurgery; TAMIS, transanal minimally invasive surgery.

A total of 681 patients (59.5% male; median age 66 (i.q.r. 59–73) years) with T1 cancer located in the rectum and with complete information on imaging staging were included (*Fig. 1*). Indications for colonoscopy included screening in 323 (47.4%), surveillance in 27 (4%), symptoms or computed tomography findings in 284 (43.9%), and other in 13 (1.9%) patients. Lesion morphology was sessile in 55.1%, pedunculated in 22.5%, and flat in 22.4% of patients. Lesions were in the upper rectum in 42.6%, middle rectum in 32.1% and lower rectum in 25.3% of patients. The mean(s.d.) size of lesions was 25.3(15.6) mm (range 3–130 mm). At baseline colonoscopy, only 53% of the lesions were suspected to contain invasive carcinoma by the endoscopist. Primary treatment was local in 540 (79.3%) lesions (endoscopy in 455 and TEM/TAMIS in 85) and oncological surgery in 141 (20.7%). A total of 203 of 455 (44.6%) patients treated primarily with endoscopy and 15 of 85 (17.6%) patients treated with primary local surgery underwent secondary oncological surgery. Overall, 298 of 681(43.7%) patients underwent oncological surgery as a primary and/or secondary treatment (*Fig. 2*). This information was collected through retrospective review of colonoscopy reports, where the endoscopist's evaluation at the time of the procedure was documented.

In total, 47 of 640 patients (7.3%) presented with an adverse oncological outcome: 14 of 360 patients treated locally presented with extraluminal recurrence and 33 of 280 patients that underwent oncological surgery had positive lymph nodes at the surgical specimen. In all, 247 of 640 patients (38.6%) could be considered as overtreated as they received unnecessary surgery whereas 14 of 640 (2.2%) could be considered undertreated. A total of 88% of all patients treated with oncological surgery had unnecessary surgery (*Fig. S1*).

The baseline characteristics depending on the type of health centre (reference centre *versus* non-reference centre) are shown in *Table S2*.

### Factors influencing the utilization of locoregional staging with MRI and/or EUS

Locoregional staging with an imaging modality was performed in 424 of 681(62.3%) patients: 234 (55.2%) with MRI only, 131(30.9%) with both MRI and EUS, and 59(13.9%) with EUS only (*Fig. S2*).

Patient and polyp characteristics in relation to the presence of imaging staging are shown in *Table 1*. The factors that were independently associated with performing locoregional staging imaging before surgery or 1 month after local treatment in those cases without surgery were T1 management in reference centre *versus* no reference centre (75.7% *versus* 24.3%; odds ratio (OR) 2.9, 95% confidence interval (c.i.) 1.5 to 5.7), location in the low and middle rectum *versus* high rectum (69.1% *versus* 30.9%; OR 3.2, 1.8 to 5.7), optical diagnostic suspicion of invasive carcinoma at baseline colonoscopy (64.8% *versus* 35.2%; OR 2.4, 1.3 to 4.5), and histology showing the presence of at least one high-risk histological feature *versus* none (69.6% *versus* 30.4%; OR 3.7, 1.8 to 7.4].

**Table 1:** Factors associated with staging (univariate and multivariate analysis)

	Total	Staging	No staging	P* (univariate)	OR (95% c.i.)	*P*† (multivariate)
No. of patients with information on staging	681	424	257			
**Sex**						
Male	405	261 of 424 (61.6%)	144 of 257 (56.0%)	0.155		
Female	276	163 of 424 (38.4%)	113 of 257 (44.0%)			
Age at diagnosis (years), mean (i.q.r.)	681	65.9 (59–73)	65.7 (59–73)	0.949		
**Reference centre**						
Yes	496	321 of 424 (75.7%)	175 of 257 (68.1%)	0.032	2.9 (1.5, 5.7)	0.002
No	185	103 of 424 (24.3%)	82 of 257 (31.9%)		1	
**Year of diagnosis**						
Before 2013	246	144 of 424 (40.0%)	102 of 257 (37.7%)	0.132		
After 2013	435	280 of 424 (66.0%)	155 of 257 (60.3%)			
**Co-morbidity (*n* = 650)**						
Mild	488	300 of 403 (74.4%)	188 of 247 (76.1%)	0.633		
Severe	276	103 of 403 (25.6%)	59 of 247 (23.9%)			
**Location (*n* = 614)**						
Low-middle rectum	353	166 of 385 (69.1%)	87 of 229 (38.0%)	< 0.001	3.2 (1.8, 5.6)	< 0.001
Upper rectum	261	119 of 385 (30.9%)	142 of 229 (62.0%)		1	
Size (*n* = 607), mean(s.d.)	681	27.4(16.9)	22.5(13.8)	0.015	NS	
**Size range (*n* = 607)**						
0–20 mm	321	168 of 359 (46.8%)	153 of 248 (61.7%)	< 0.001	NS	
> 20 mm	286	191 of 359 (53.2%)	95 of 248 (38.3%)			
**Morphology (*n* = 512)**						
Pedunculated	116	43 of 299 (14.4%)	73 of 213 (34.3%)	< 0.001	NS	
Non-pedunculated	396	256 of 299 (85.6%)	140 of 213 (65.7%)			
**Suspicion of invasive carcinoma (*n* = 600)**						
Yes	320	245 of 378 (64.8%)	75 of 222 (33.8%)	< 0.001	2.4 (1.3, 4,5)	0.006
No	280	133 of 378 (35.2%)	147 of 222 (66.2%)		1	
**Indication for baseline colonoscopy (*n* = 647)**						
Screening	363	214 of 394 (54.3%)	149 of 253 (58.9%)	0.253		
Symptoms/CT findings	284	180 of 394 (45.7%)	104 of 253 (41.1%)			
**Oncological surgery as final treatment (*n* = 681)**						
Yes	298	206 of 424 (48.6%)	92 of 257 (35.8%)	< 0.001	NS	
No	383	218 of 424 (51.4%)	165 of 257 (64.2%)			
**High-risk histological factors + (*n* = 609)**						
Yes	403	265 of 381 (69.5%)	138 of 228 (60.5%)	0.023	3.7 (1.8, 7.4)	< 0.001
No	206	116 of 381 (30.4%)	90 of 228 (39.5%)		1	
**N positive at surgery or distant recurrence (*n* = 654)**						
Yes	60	45 of 410 (11.0%)	15 of 244 (6.1%)	0.039	NS	
No	594	365 of 410 (89.0%)	229 of 244 (93.9%)			

Values are *n* (%) unless otherwise stated. OR, odds ratio; c.i., confidence interval; s.d., standard deviation; NS, non-significant. * Univariate analysis (Chi-square test for categorical variables; Student's t-test or Mann–Whitney *U* test for continuous variables, as appropriate). Statistically significant values (P < 0.05); † Multivariable analysis performed using binary logistic regression.

See *Tables S3*, *S4* for factors associated with EUS and MRI use, respectively.

### Diagnostic accuracy of imaging staging modalities

#### MRI and/or EUS for T staging

EUS and MRI correctly diagnosed T stage in 69 of 117 (59%) and 54 of 191 (28.3%) T1 rectal cancers, respectively. When combined (MRI and/or EUS), T stage was correctly diagnosed in 76 of 231 (32.9%) of patients and overstaged in 155 of 231 (67.1%).

The factors independently associated with correct T staging by MRI and/or EUS were size ≥ 20 mm, suspicion of invasive carcinoma, and referral centres (*Table S2*). Interestingly, MRI T staging was more often correct in referral centres than non-referral centres (46 of 139 (33.1%) *versus* 8 of 52 (15.4%); *P* < 0.02, respectively) whereas EUS correctly diagnosed T stage in 62 of 100 (62%) *versus* 7 of 174 (1.2%) (*P* = 0.106) in referral and non-referral centres, respectively.

#### MRI and/or EUS for N staging

For patients undergoing locoregional staging with MRI and/or EUS and treated with oncological surgery, N staging was correct (MRI and EUS) in 103 of 132 (78%) patients. Remarkably, MRI correctly diagnosed N stage in 71 of 83 (85.5%) *versus* 27 of 39 (69.2%) (*P* = 0.049) patients in referral and non-referral centres, respectively, whereas EUS correctly diagnosed N stage in 40 of 44 (90.9%) *versus* 4 of 5 (80%) (*P* = 0.43) patients in referral and non-referral centres, respectively.

*Table 2* shows the accuracy of MRI + EUS, MRI alone, EUS alone, and MRI and/or EUS. In those patients that underwent a lymphadenectomy (oncological surgery), pathological N staging was used as the standard, whereas adverse oncological outcome was used as the standard for the whole cohort.

**Table 2:** Diagnostic accuracy for N staging with MRI and/or EUS, MRI + EUS, MRI only, and EUS only

	MRI and/or EUS		MRI + EUS		MRI		EUS	
	N+ or recurrence		N+ or recurrence		N+ or recurrence		N+ or recurrence	
	Whole cohort (*n* = 262)	Patients with oncological surgery	Whole cohort (*n* = 82)	Patients with oncological surgery	Whole cohort (*n* = 243)	Patients with oncological surgery	Whole cohort (*n* = 121)	Patients with oncological surgery
Sensitivity	3 of 23 (13; 3.4, 34.7%)	2 of 13 (15.4; 1.9, 46.3%)	0 of 2 (0.0; 0.0, 80.2%)	0 of 1 (0.0; 0.0, 94.0%)	3 of 19 (15.8; 4.2, 40.5%)	2 of 12 (16.7; 2.9, 49.1%)	0 of 6 (0.0; 0.0, 48.0%)	0 of 2 (0.0; 0.0, 80.0%)
Specificity	216 of 239 (90.4; 85.7, 93.7%)	90 of 109 (82.6; 73.9, 88.9%)	70 of 80 (87.5; 77.8, 93.5%)	30 of 38 (78.9; 62.2, 89.9%)	204 of 224 (91.1; 86.3, 94.3%)	94 of 110 (85.4; 77.1, 91.2%)	111 of 115 (96.5; 90.8, 98.9%)	44 of 47 (93.6; 81.4, 98.3%)
PPV	3 of 26 (11.5; 3.0, 31.3%)	2 of 21 (9.5; 1.7, 31.8%)	0 of 10 (0.0; 0.0, 34.4%)	0 of 8 (0.0; 0.0, 40.2%)	3 of 23 (13.0; 3.4, 34.7%)	2 of 18 (11.1; 1.9, 36.0%)	0 of 4 (0.0; 0.0, 60.0%)	0 of 3 (0.0; 0.0, 69.0%)
NPV	216 of 236 (91.5; 87.0, 94.6%)	90 of 101 (89.1; 80.9, 94.2%)	70 of 72 (97.2; 89.4, 99.5%)	30 of 31 (96.8; 81.5, 99.8%)	204 of 220 (92.7; 88.2, 95.6%)	94 of 104 (90.4; 82.6, 95.0%)	111 of 117 (94.9; 88.7, 97.9%)	44 of 46 (95.6; 83.9, 99.2%)

Values are *n* (%; 95% confidence interval). EUS, endoscopic ultrasound; MRI, magnetic resonance imaging; PPV, positive predictive value; NPV, negative predictive value.

### MRI and EUS concordance for T and N staging and comparison between diagnostic accuracy for N staging

Among the 131 patients who underwent both MRI and EUS for T staging, discrepancies between the two methods were common. In 27 (21%) patients, both MRI and EUS provided incorrect results. EUS was accurate and MRI was incorrect in 23 (17.5%) patients, whereas both modalities provided correct T staging in 19 (14.5%) patients. Only four (3%) patients had correct staging with MRI but incorrect staging with EUS.

For N staging, among the 131 patients, 39 had known N staging confirmed by histology from surgery, serving as the standard. Among these, 30 (76.9%) patients had correct results from both MRI and EUS. Incorrect results from both modalities occurred in only one (2.6%) patient. EUS was accurate and MRI was incorrect in five (12.8%) patients, whereas MRI was accurate and EUS incorrect in three (7.7%) patients (*Table S5*).

The specificity for detecting LNM was high and comparable between the two modalities: 93.6% for EUS and 85.4% for MRI (*P* = 0.479). However, a comparison of sensitivity was not feasible because only one patient with positive lymph nodes in the surgical specimen was reported as negative by both imaging methods.

### Oncological adverse outcome and staging

Sixty-one of 681 patients (9%) included in the analyses presented with an oncological adverse outcome during follow-up: 34 of 298 that underwent surgery had a positive lymph node in the surgical specimen; 15 of 383 that underwent local treatment presented an extraluminal recurrence, and 12 patients that underwent oncological surgery with negative lymph nodes in the surgical specimen presented with extraluminal recurrence during follow-up.

Factors independently associated to oncological adverse outcomes were suspicion of invasiveness at basal colonoscopy *versus* no suspicion (75% *versus* 25%; OR 2.4, 95% c.i. 1.2 to 4.7) and presence of at least one high-risk histological feature *versus* no high-risk histological features (89% *versus* 11%; OR 4.3, 1.8 to 10.4).

**Fig. 3:**
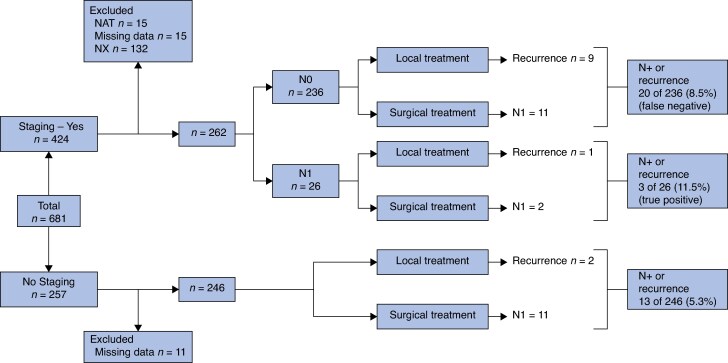
Oncological outcome of patients in relation to staging and treatment NAT, neoadjuvant therapy.

*Figure 3* displays the oncological outcomes of patients in relation to the staging and the treatment option, after excluding the ones that underwent NAT, had missing data, and were Nx. Interestingly, 20 of 236 (8.5%) patients with N0 staging at MRI and/or EUS had an adverse oncological outcome (false negative), whereas only 3 of 26 (11.5%) with N+ staging presented with an adverse oncological outcome (true positive). Only 13 of 246 (5.3%) patients that did not have staging had an oncological adverse outcome.

## Discussion

This large, multicentre cohort study highlights critical gaps in the management of early rectal cancer, focusing on the underperformance of imaging modalities and the variability in real-world clinical practice. Despite theoretical advantages of locoregional staging, the present study’s findings indicate substantial limitations in accuracy and practical implementation, particularly concerning endoscopic techniques and imaging methods such as EUS and MRI.

Factors influencing imaging locoregional staging underline the imperative for multidisciplinary consensus and guidelines to ensure standardized clinical care. In practice, modality selection was driven by tumour location (lower/mid-rectum), macroscopic suspicion of invasion, and management at reference centres. Both EUS and MRI exhibited suboptimal accuracy for T staging, with a trend of overstaging, whereas they showed higher accuracy for N staging; diminished sensitivity and positive predictive value raise concerns. The retrospective design and heterogeneity in rectal cancer management during the study period may have influenced these results. Notably, the observed low adherence to guidelines, where up to 55% of patients were staged using MRI contrary to the 2013 ESGAR guidelines^23^ recommending EUS for local-regional staging, underscores a need for heightened awareness and improved implementation of recommended techniques.

In contrast to current recommendations favouring MRI for early rectal cancer staging^3,7,9,10^, the results support the use of EUS over MRI for T staging. EUS demonstrated twice the accuracy of MRI, irrespective of healthcare centre type. Importantly, EUS outperformed MRI in distinguishing benign polyps from early invasive cancers, addressing a critical aspect of real-life practice. The tendency for MRI to overestimate the depth of invasion in early tumours should be acknowledged, given its impact on therapeutic decisions.

In early rectal cancer, accurate locoregional staging is vital to guide therapeutic strategies, particularly in ruling out lymph node involvement. However, this study demonstrates that, despite staging, almost 90% of T1 CRC cases were overtreated, and lymph node removal did not prevent recurrence. This complexity underscores the need for a multidisciplinary approach considering technical aspects and risk-–benefit balance based on oncological outcomes. Current criteria for N+ may not be suitable for T1 tumours, potentially excluding patients from curative total mesorectal excision. Sensitivity of imaging techniques for detecting microscopic metastases in T1 lymph nodes may differ from advanced cancer, raising questions about criteria applicability^24^. Considering the limitations of staging techniques, the authors advocate for a stepwise approach, with local *en bloc* resection as the initial step when T1 rectal cancer is suspected in a polyp. Locoregional staging should be performed in case of suspicion of deep invasive cancer.

Despite the retrospective nature of the study introducing bias, the large nationwide cohort offers valuable clinical information, forming a foundation for future prospective trials. Unlike clinical trials that often include lesions with gross morphologies suggestive of invasive cancer, this cohort reflects real-world practice, where a significant proportion of T1 cases are primarily treated with endoscopy as they arise in normal-appearing polyps (the so called ‘oops lesions’)^25^. The differences in case profiles highlight the need for research and consensus protocols specifically focused on managing T1 CRC. The benefit of endoscopic resection in these cases lies in precise histological evaluation and potential curative resection of residual tumours at the scar site, mitigating risks of local recurrence.

This study emphasizes the limitations of EUS and MRI in real-world clinical settings for T1 rectal cancer staging. Whereas EUS showed better diagnostic accuracy than MRI for T staging, both methods tended to overstage. The high specificity of EUS and MRI for N staging is counterbalanced by low sensitivity, raising concerns about potential overtreatment. The findings advocate for a stepwise diagnostic approach, beginning with endoscopic resection for suspected T1 cancers, followed by locoregional staging as needed.

Comprehensive training and standardized protocols across diverse clinical settings are essential in improving care for patients with early rectal cancer. These efforts will support the integration of advanced endoscopic techniques into routine practice, aligning with the overarching goal of organ preservation and improved patient outcomes.

Emerging evidence on neoadjuvant immunotherapy (particularly in dMMR/MSI-H rectal cancer) is expanding organ-preserving pathways, further elevating the stakes of accurate locoregional staging to identify candidates and avoid unnecessary radical surgery.

With acknowledgement of the likely underestimation of current MRI accuracy due to the study's retrospective nature, the importance of implementing new guidelines and evidence to enhance MRI accuracy in routine clinical settings is emphasized. Ongoing training and adherence to reliable protocols among radiologists, not confined to academic centres, are crucial to align with best practices^26,27^.

## Collaborators

Member of the EpiT1 Consortium: G. Casanova (Hospital Clinic of Barcelona, Barcelona, Spain); O. Murcia (Hospital General Universitario de Alicante, Valencia, Spain); M. Jimeno (Germans Trias i Pujol University Hospital, Badalona, Spain); N. Ascon (Althaia Xarxa Assistencial Universitària de Manresa, Manresa, Spain); F. Balaguer (Hospital Clinic of Barcelona, Clinic Institute of Digestive and Metabolic Disease (ICMDIM) of Barcelona, Barcelona, Spain. Facultat de Medicina i Ciències de la Salud, Universitat de Barcelona (UB), Barcelona, Spain).

## Supplementary Material

zrag003_Supplementary_Data

## Data Availability

The entire database is available upon request to interested researchers from the corresponding author.
