# Supplementary material for: Accuracy and variability of locoregional staging in T1 rectal cancer: nationwide multicentre cohort study
Source: BJS Open. 2026 Mar 31;10(2):zrag003. doi: 10.1093/bjsopen/zrag003 (PMC13036738; doi:10.1093/bjsopen/zrag003)
Supplement: zrag003_Supplementary_Data [file zrag003_supplementary_data.docx]

**Accuracy and Variability of Locoregional Staging in T1 Rectal Cancer: A Nationwide Multicentre Cohort Study**

Maria Daca-Alvarez, MD ^1^; Cristina Manzotti, MD^2^; Diana Zaffalon, MD^3^; Isabel Portillo, MD, PhD^4^; Luis Bujanda, MD, PhD^5^; Inés Gil-Lasa, MD^5^, Gemma Ibañez-Sanz, MD, PhD^6,7^; Xavier Sanjuan,MD,PhD^7^; Alberto Herreros-de-Tejada, MD, PhD^8^; Inmaculada Salces, MD^9^; Lara Aguilera, MD^10^; Marta Ponce, MD, PhD^11^; Ángeles Pizarro, MD, PhD^12^; David Barquero, MD, PhD^13^; Ignasi Puig, MD, PhD^14,15,16^; Pilar Diez Redondo, MD, PhD^17^; Fernando Martínez de Juan, MD,PhD^18^; Victor Jair Morales, MD^19^; Marco Alburquerque, MD^20^; Salvador Machlab, MD, PhD^21^; Angel Ferrandez, MD, PhD^22^; Beatriz Peñas, MD^23^; Alvaro Díaz-González, MD, PhD^24^; Lluïsa Sargatal, MD^3^; Rodrigo Jover, MD, PhD^25^; Luis Hernandez, MD^26^; Alberto Pérez Pedrosa, MD^27^; Eva Musulen, MD, PhD^28^; Goretti Hernandez, MD, PhD^29^; Marita Trelles, MD^30^; Akiko Ono, MD, PhD^31^; Jorge Lopez Vicente, MD^32^; Raquel Bravo, MD^33^ ; Juan R Ayuso, MD, PhD^34^; Angels Ginés, MD, PhD ^1,35^; Karmele Saez de Gordoa, MD^36^; Miriam Cuatrecasas MD, PhD^35,36^; Maria Pellisé MD, PhD^1,35^. **EpiT1 Consortium**

^1^ Gastroenterology Department,Hospital Clinic of Barcelona, Clinic Institute of digestive and Metabolic disease (ICMDIM) of Barcelona, Spain; Centro de Investigación Biomédica en Red en Enfermedades Hepáticas y Digestivas (CIBEREHD), Institut d'Investigacions Biomédiques August Pi i Sunyer, Barcelona, Spain; ^2^ Gastroenterology and Digestive Endoscopy Unit, Azienda USL-IRCCS Reggio nell'Emilia, Reggio Emilia, Italy; ^3^Gastroenterology Department, Consorci Sanitari de Terrassa, Barcelona,Spain; ^4^Dirección General de Osakidetza-Servicio Vasco de Salud. Instituto de Investigación BioBizkaia, Spain; ^5^Department of Gastroenterology. BioGipuzkoa Health Research Institute. Centro de Investigación Biomédica en Red de Enfermedades Hepáticas y Digestivas (CIBERehd). Universidad del País Vasco (UPV/EHU). San Sebastián, Spain; ^6^ICO Institut Català d'Oncologia; ^7^Hospital Universitari de Bellvitge. ONCOBELL Program, Bellvitge Biomedical Research Institute (IDIBELL), Barcelona, Spain; ^8^Department of Gastroenterology, Puerta De Hierro University Hospital, Madrid, Spain; ^9^Hospital Universitario 12 de Octubre, Madrid,Spain; ^10^Hospital Vall D'Hebron, Barcelona, Spain; ^11^Hospital Clinico Valencia, Valencia, Spain; ^12^Hospital Universitario Virgen del Rocío,Sevilla, Spain; ^13^Hospital Sant Joan Despí Moisès Broggi; ^14^Digestive Diseases Department, Althaia Xarxa Assistencial Universitària de Manresa, 08243 Manresa, Spain; ^15^Facultat de Medicina, Universitat de Vic-Central de Cataluña (UVIC-UCC), 08500 Vic, Spain; ^16^Gastrointestinal Oncology Endoscopy and Surgery (GOES) research group, Institut de Recerca i Innovació en Ciències de la Vida i de la Salut a la Catalunya Central (IRIS-CC), 08500 Vic, Spain; ^17^Hospital Universitario Río Hortega, Valladolid, Spain; ^18^Instituto Valenciano de Oncología (IVO), Valencia, Spain; ^19^Hospital General de Granollers,Barcelona,Spain; ^20^Hospital de Palamós,Girona, Spain; ^21^Digestive Endoscopy Unit. Institut d‘Investigació i Innovació Parc Taulí I3PT, Parc Taulí Hospital Universitari, Sabadell, Spain. Departament de Medicina. Universitat Autónoma de Barcelona; ^22^Hospital Clínico Universitario Lozano Blesa,Zaragoza,Spain; ^23^Hospital Universitario Ramón y Cajal,Madrid,Spain; ^24^Gastroenterology and Hepatology Department, Clinical and Translational Research in Digestive Diseases Group, Valdecilla Research Institute (IDIVAL), Marqués de Valdecilla University Hospital, Santander, Spain; ^25^Servicio de Medicina Digestiva. Hospital General Universitario Dr. Balmis. Instituto de Investigación Sanitaria ISABIAL. Departamento de Medicina Clínica. Universidad Miguel Hernández. Alicante; ^26^Hospital Santos Reyes Aranda Duero,Burgos,Spain; ^27^Complexo Hospitalario de Ourense; ^28^Hospital Univeritari General de Catalunya-Grupo Quironsalud; Insitut de Recerca contra la Leucèmia Josep Carreras; ^29^Servicio de Aparato Digestivo, Hospital Universitario de Canarias,Santa Cruz de Tenerife,Spain; ^30^Hospital de Inca,Islas Baleares,Spain; ^31^Hospital Virgen de la Arrixaca,Murcia, Spain; ^32^Hospital Universitario de Móstoles,Madrid, Spain; ^33^Department of colorectal surgery , Hospital Clínic of Barcelona,Spain; ^34^Radiology Department - CDI, Hospital Clínic of Barcelona,Spain; ^35^Facultat de Medicina i Ciències de la Salud, Universitat de Barcelona (UB), Barcelona, Spain; ^36^Department of Pathology, center of biomedical diagnosis (CDB) , Hospital Clínic of Barcelona

**Corresponding author contact information:**

Maria Pellisé, MD, PhD. Department of Gastroenterology, Hospital Clinic of Barcelona, Villaroel 170, 08036 Barcelona, Spain (e-mail: [mpellise@ub.edu](mailto:mpellise@ub.edu))

**Supplementary material –Index**

| **Supplementary Figures** |  |
| --- | --- |
| *Suplemmentary Figure 1: Oncological adverse outcome of the cohort* | *page 4* |
| *Suplemmentary Figure 2: Staging modality of T1 rectal cancer* | *page 5* |

| **Supplementary Tables** |  |
| --- | --- |
| *Supplementary Table 1. Baseline characteristic of the cohort between reference and non-reference center* | *page 6* |
| *Supplementary Table 2: Factors associated with correct T staging  (Univariate and Multivariate analysis)* | *page 7* |
| *Supplementary Table 3: Factors associated with the use of endoscopic ultrasound ( EUS) for logoregional staging in T1 rectal cancer (Univariate and Multivariate analysis)* | *page 8* |
| *Supplementary Table 4: Factors associated with the use of Magnetic resonance imaging ( MRI) for locoregional staging in T1 rectal cancer  (Univariate and Multivariate analysis)* | *page 9* |

| *Supplementary Table 5. Concordance for N Staging of MRI and EUS in those patients undergoing both tests and in relation with pathology in surgical specimen* | *page 10* |
| --- | --- |

| **Supplementary Appendixes** |  |
| --- | --- |
| *Supplementary Appendix 1.* STROBE Statement: Checklist of items that should be included in reports of observational studies. | *page 11-12* |

**SUPPLEMENTARY FIGURES**

**Suplemmentary Figure 1: Oncological adverse outcome of the cohort**

**Suplemmentary Figure 2: Staging modality of T1 rectal cancer**


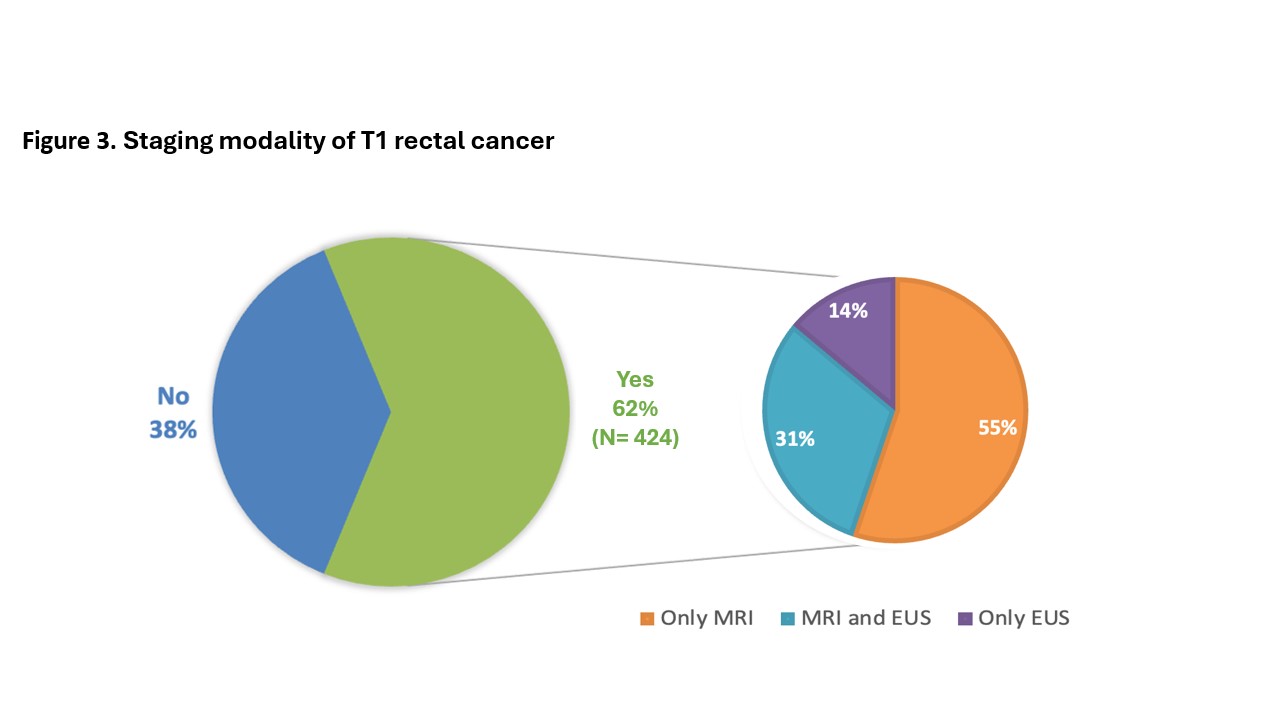


**SUPPLEMENTARY TABLES**

**Supplementary Table 1. Baseline characteristic of the cohort between reference and non-reference center**

**
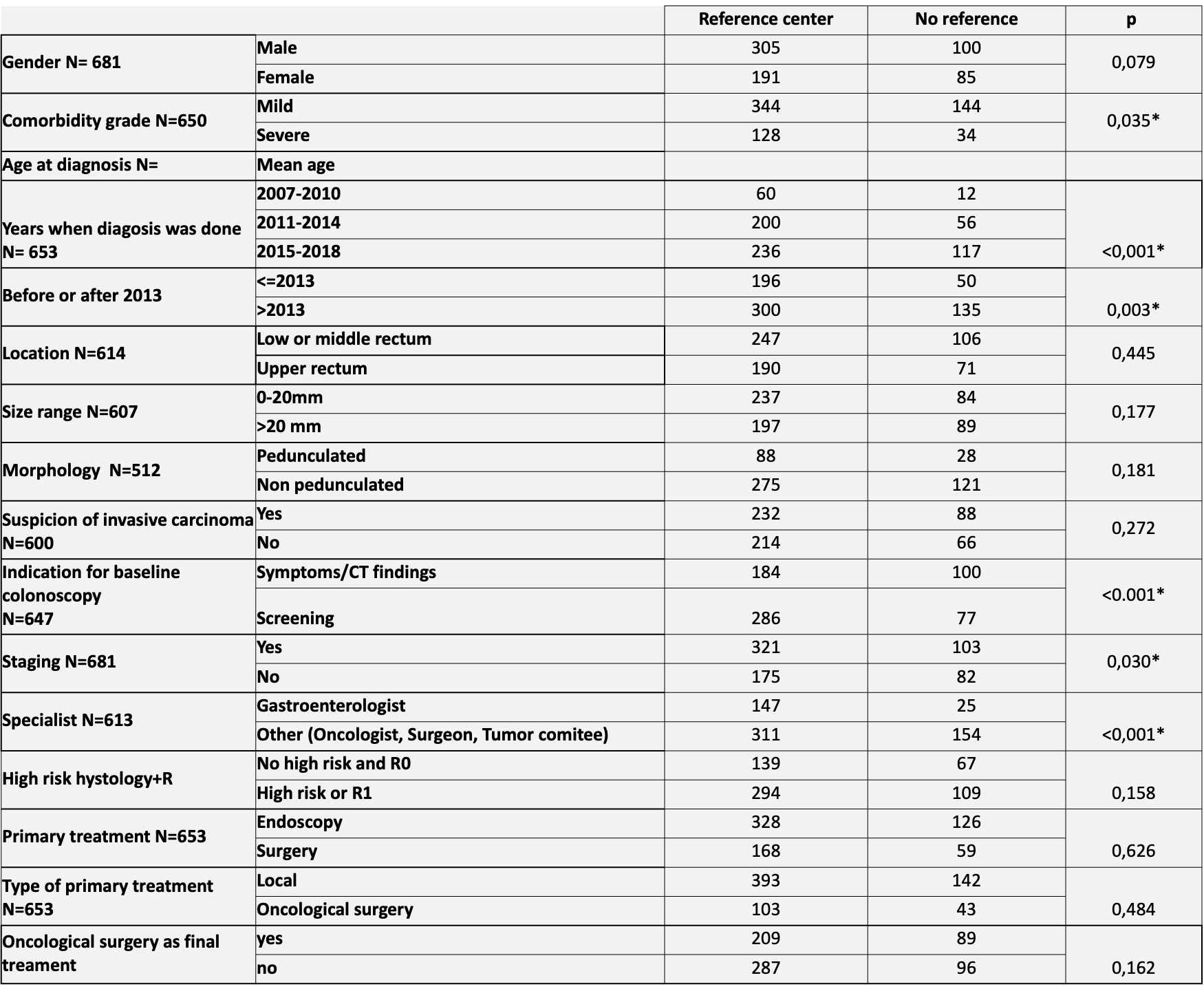
**

**Supplementary Table 2: Factors associated with correct T staging  (Univariate and Multivariate analysis)**


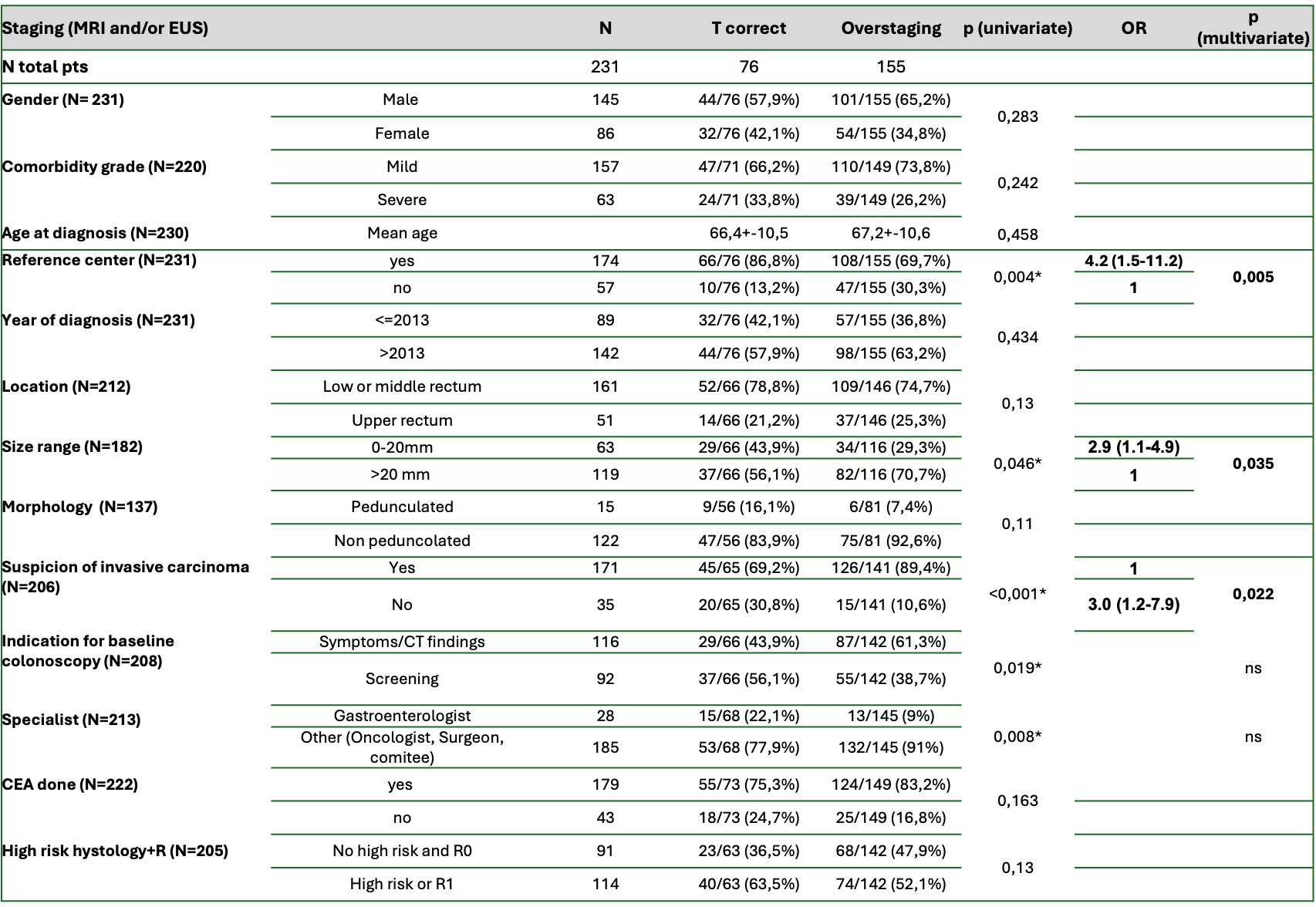


**Supplementary Table 3: Factors associated with the use of endoscopic ultrasound (EUS) for logoregional staging in T1 rectal cancer (Univariate and Multivariate analysis)**

|  |  | **EUS – YES**  **N=190** | **EUS – NO**  **N=488** | **pº** | **OR** | **pª** |
| --- | --- | --- | --- | --- | --- | --- |
| **Gender N=678** | **Male** | 120 (63,2%) | 283 (58%) | 0,229 | - |  |
|  | **Female** | 70 (36,8%) | 205 (42%) |  |  |  |
| **Age at diagnosis N=678** | **Mean age** | 66,5 +9,6 | 65,5 +10,0 | 0,265 | - |  |
| **Years when diagosis was done N=677** | **2007-2010** | 32 (16,8%) | 40 (8,2%) | 0,002* | NS |  |
|  | **2011-2014** | 74 (38,9%) | 182 (37,4%) |  |  |  |
|  | **2015-2018** | 84 (44,2%) | 265 (54,4%) |  |  |  |
| **Comorbidity N= 650** | **Mild** | 128 (68,8%) | 357 (77,4%) | 0,022* | NS |  |
|  | **Severe** | 58 (31,2%) | 104 (22,6%) |  |  |  |
| **Location N = 614** | **Low-middle rectum** | 128 (76,6%) | 222 (50,1%) | <0,001* | 2,9 (1,7-5,1) | <0,001 |
|  | **Upper rectum** | 39 (23,4%) | 221 (49,9%) |  | 1 |  |
| **Size N=607** | **Mean +/- SD** | 28,0 +16,3 | 24,5 +15,7 | 0,020* | NS |  |
| **Size range N=607** | **0-20mm** | 66 (43,1%) | 253 (56%) | 0,006* | 1 | 0,049 |
|  | **>20 mm** | 87 (56,9%) | 199 (44%) |  | 1,7 (1,01-2,78) |  |
| **Morphology N=512** | **Pedunculated** | 16 (13%) | 100 (25,7%) | 0,003* | NS |  |
|  | **Non peduncolated** | 107 (87%) | 289 (74,3%) |  |  |  |
| **Suspicion of invasive carcinoma N=600** | **Yes** | 114 (68,7%) | 205 (47,3%) | <0,001* | NS |  |
|  | **No** | 52 (31,3%) | 228 (52,7%) |  |  |  |
| **Indication for baseline colonoscopy N=647** | **Screening** | 84 (49,7%) | 199 (41,8%) | 0,076 | - |  |
|  | **Symptoms/CT findings** | 85 (50,3%) | 277 (58,2%) |  |  |  |
| **Specialist N=637** | **Gastroenterologist** | 21 (11,7%) | 151 (33,3%) | <0,001* | 1 | 0,037 |
|  | **Other (Oncologist, Surgeon, Tumor comitee)** | 158 (88,3%) | 303 (66,7%) |  | 2,1 (1,04-4,11) |  |
| **Oncological surgery as final treatment?** | **Yes** | 89 (46,8%) | 204 (41,9%) | 0,243 |  |  |
|  | **no** | 101 (53,2%) | 283 (58,1%) |  |  |  |

**º Univariate analysis**

**ª Multivariate analysis**

**Supplementary Table 4: Factors associated with the use of Magnetic resonance imaging ( MRI) for locoregional staging in T1 rectal cancer  (Univariate and Multivariate analysis)**

|  |  | **MRI – YES**  **N= 365** | **MRI - NO**  **N= 310** | **pº** | **OR (95% CI)** | **pª** |
| --- | --- | --- | --- | --- | --- | --- |
| **Gender-N=681** | Male | 221 (60,5%) | 182 (58,7%) | 0,628 | - |  |
|  | Female | 144 (39,5%) | 128 (41,3%) |  |  |  |
| **Age at diagnosis- N=680** | Mean age | 65,8+9,9 | 65,8+9,9 | 0,989 | - |  |
| **Years when diagosis was done** | 2007-2010 | 32 (8,8%) | 37 (11,9%) | 0,114 | - |  |
|  | 2011-2014 | 130 (35,6%) | 124 (40%) |  |  |  |
|  | 2015-2018 | 203 (55,6%) | 149 (48,1%) |  |  |  |
| **Comorbidity N= 644** | Mild | 259 (75,3%) | 227 (75,7%) | 0,912 | - |  |
|  | Severe | 85 (24,7%) | 73 (24,3%) |  |  |  |
| **Location N = 610** | Low-middle rectum | 230 (68,9%) | 120 (43,5%) | <0,001* | 2,4 (1,5-3,8) | <0,001 |
|  | Upper rectum | 104 (31,1%) | 156 (56,5%) |  | 1 |  |
| **Size N=607** | Mean +/- SD | 27,4+-17,0 | 23,3+-14,3 | 0,001* | NS |  |
| **Size range N=607** | 0-20mm | 148 (47,1%) | 171 (59%) | 0,004* | NS |  |
|  | >20 mm | 166 (52,9%) | 119 (41%) |  |  |  |
| **Morphology N=512** | Pedunculated | 38 (14,6%) | 78 (31%) | <0,001* | 1 | 0,024 |
|  | Non pedunculated | 222 (85,4%) | 174 (69%) |  | 1,9 (1,1-3,5) |  |
| **Suspicion of invasive carcinoma N=600** | Yes | 218 (66,7%) | 102 (37,5%) | <0,001* | 2,6 (1,7-4,2) | <0,001 |
|  | No | 109 (33,3%) | 170 (62,5%) |  | 1 |  |
| **Indication for baseline colonoscopy N=647** | Screening | 153 (44,6%) | 130 (43%) | 0,69 | - |  |
|  | Symptoms/CT findings | 190 (55,4%) | 172 (57%) |  |  |  |
| **Specialist N=637** | Gastroenterologist | 58 (16,9%) | 114 (39,7%) | <0,001* | 1 | 0,002 |
|  | Other (Oncologist, Surgeon, Tumor comitee) | 286 (83,1%) | 173 (60,3%) |  | 2,3 (1,4-3,9) |  |
| **Oncological surgery as final treatment?** | Yes | 185 (50,7%) | 110 (35,5%) | <0,001* | NS |  |
|  | No | 180 (49,3%) | 200 (64,5%) |  |  |  |

**º Univariate analysis**

**ª Multivariate analysis**

**Supplementary Table 5. Concordance for N Staging of MRI and EUS in those patients undergoing both tests and in relation with pathology in surgical specimen**

|  | **For N+ (Sensitivity)** | | | **For N0 (Specificity)** | | |
| --- | --- | --- | --- | --- | --- | --- |
|  | **MRI Positive** | **MRI  Negative** | **Total** | **MRI Positive** | **MRI Negative** | **Total** |
| **EUS Positive** | 0 | 0 | 0 | 0 | 3 | 3 |
| **EUS Negative** | 0 | 1 | 1 | 5 | 30 | 35 |
| **Total** | 0 | 1 | 1 | 5 | 33 | 38 |

**SUPPLEMENTARY APPENDIXES**

**Supplementary Appendix 1.  STROBE Statement: Checklist of items that should be included in reports of observational studies.**

|  | Item No | Recommendation | Page No |
| --- | --- | --- | --- |
| **Title and abstract** | 1 | (*a*) Indicate the study’s design with a commonly used term in the title or the abstract |  |
|  |  | (*b*) Provide in the abstract an informative and balanced summary of what was done and what was found | 1-3 |
| Introduction | | | |
| Background/rationale | 2 | Explain the scientific background and rationale for the investigation being reported | 4-5 |
| Objectives | 3 | State specific objectives, including any prespecified hypotheses | 4-5 |
| Methods | | | |
| Study design | 4 | Present key elements of study design early in the paper | 6-7 |
| Setting | 5 | Describe the setting, locations, and relevant dates, including periods of recruitment, exposure, follow-up, and data collection | 6-7 |
| Participants | 6 | (*a*) Give the eligibility criteria, and the sources and methods of selection of participants. Describe methods of follow-up | 6-7 |
|  |  | (*b*) For matched studies, give matching criteria and number of exposed and unexposed |  |
| Variables | 7 | Clearly define all outcomes, exposures, predictors, potential confounders, and effect modifiers. Give diagnostic criteria, if applicable | 6-7 |
| Data sources/ measurement | 8* | For each variable of interest, give sources of data and details of methods of assessment (measurement). Describe comparability of assessment methods if there is more than one group | 6-7 |
| Bias | 9 | Describe any efforts to address potential sources of bias | 6-7 |
| Study size | 10 | Explain how the study size was arrived at | 6-7 |
| Quantitative variables | 11 | Explain how quantitative variables were handled in the analyses. If applicable, describe which groupings were chosen and why | 6-7 |
| Statistical methods | 12 | (*a*) Describe all statistical methods, including those used to control for confounding | 6-7 |
|  |  | (*b*) Describe any methods used to examine subgroups and interactions |  |
|  |  | (*c*) Explain how missing data were addressed |  |
|  |  | (*d*) If applicable, explain how loss to follow-up was addressed |  |
|  |  | (*e*) Describe any sensitivity analyses |  |
| Results | | |  |
| Participants | 13* | (a) Report numbers of individuals at each stage of study—eg numbers potentially eligible, examined for eligibility, confirmed eligible, included in the study, completing follow-up, and analysed | 8-10 |
|  |  | (b) Give reasons for non-participation at each stage |  |
|  |  | (c) Consider use of a flow diagram |  |
| Descriptive data | 14* | (a) Give characteristics of study participants (eg demographic, clinical, social) and information on exposures and potential confounders | 8-10 |
|  |  | (b) Indicate number of participants with missing data for each variable of interest |  |
|  |  | (c) Summarise follow-up time (eg, average and total amount) |  |
| Outcome data | 15* | Report numbers of outcome events or summary measures over time | 8-10 |

| Main results | 16 | (*a*) Give unadjusted estimates and, if applicable, confounder-adjusted estimates and their precision (eg, 95% confidence interval). Make clear which confounders were adjusted for and why they were included |  |
| --- | --- | --- | --- |
|  |  | (*b*) Report category boundaries when continuous variables were categorized |  |
|  |  | (*c*) If relevant, consider translating estimates of relative risk into absolute risk for a meaningful time period | 8-10 |
| Other analyses | 17 | Report other analyses done—eg analyses of subgroups and interactions, and sensitivity analyses | 8-10 |
| Discussion | | | |
| Key results | 18 | Summarise key results with reference to study objectives | 11-12 |
| Limitations | 19 | Discuss limitations of the study, taking into account sources of potential bias or imprecision. Discuss both direction and magnitude of any potential bias | 11-12 |
| Interpretation | 20 | Give a cautious overall interpretation of results considering objectives, limitations, multiplicity of analyses, results from similar studies, and other relevant evidence | 11-12 |
| Generalisability | 21 | Discuss the generalisability (external validity) of the study results | 11-12 |
| Other information | | | |
| Funding | 22 | Give the source of funding and the role of the funders for the present study and, if applicable, for the original study on which the present article is based | 2 |

*Give information separately for exposed and unexposed groups.
